# Appearance in Buffalo of Cerebro-Spinal Meningitis

**Published:** 1872-03

**Authors:** 


### Editorial.

### Appearance in Buffalo, of Cerebro-Spinal Meningitis.

Within the past few weeks, Cerebro-Spinal Meningitis has made its first appearance in Buffalo and near vicinity, at least, in sufficient frequency to constitute anything like epidemic form; sporadic cases may have been observed and recognized, or passed under other name. The symptoms are varied and numerous, mostly pointing to serious disturbance of the great nervous centre, but thus far, these symptoms have been in many cases quite equivocal, and not unfrequently so feebly marked, as to leave doubt as to the nature of the malady. We have formerly, as now, been treating patients with similar symptoms, and but for the fact of greater frequency, might overlook the peculiarities of the cases, which we now regard as inflamation of the membranes of the brain and spinal cord, and call "Cerebro-Spinal Meningitis," meaning that epidemic disease which has appeared anew in this country, within the last five or six years.

"Spotted Fever," is a name which seems to us wholly inappropriate, and which conveys no rational idea of the nature or location of the disease, and the reasons assigned for ever giving it this name, are entirely inadiquate; "Inflammation of the Membranes of the Brain and Spinal Cord," may be made to mean something in intelligent families; but as there are no "spots" either before or after death, every one is ready to inquire, why call it Spotted Fever? Petechial spots may doubtless occur in this as in all low forms of disease, but if present, they are the result of a condition common to all diseases of low type, and do not diserve to give name to such malady.

It may be interesting to some of our readers if we relate the principal symptoms present in the cases thus far observed, as there has been great differences noticed in different cases, and in different epidemics, in different places.

Some cases commence by a sudden attack, the patient being noticed at first, as having some grave malady; other cases the access is more gradual, the disease not being established and recognizable under three or four days. Pain, and especially pain in the head is one of the first and most constant symptoms of an attack. It is increased by motion, and is generally referred to the neck and head. Pressure does not generally increase the pain referred to back and neck, but as most of the cases have been in children, it has not been easy to determine the amount or location of pain; pain in other parts of body is often complained of. Vomiting is often present from the outset of the disease. Delirium comes on early in severe cases and partial or complete unconsciousness is often soon added; a peculiar unconscious, frequently repeated scream, thought by attendants to indicate pain, has been an unpleasant symptom in a few instances.

Oscillation and turning upwards of the eye-ball is an almost constant symptom; the pupil varying, at times dilated, again normal or contracted.

Tonic contraction of the muscles of the neck has been noticed in most well marked cases and in some instances a complete general convulsion is an early symptom. The urinary and alvine secretions normal so far as common observation extended, no special examinations having been made.

The respirations are sometimes increased, but in many cases not obviously changed. Bronchitis, with hurried respiration and cough appears frequently as a complication or as a consequence of the disturbance of nerve action. Pulse varies greatly in the same case at different times. At first it may not be changed, it may be some slower than natural or it may be very rapid, 160 per minute; it is often noticed as irregular. The heat of the surface is also liable to the same variations.

The cases thus far observed have either died early, within a few days, or appear slowly recovering, no permanent disabilities. No deafness, or blindness or paralysis resulting. The proportion of deaths to cases cannot yet be determined accurately, but it seems probable that recovery is the rule, and that nearly all cases which survive the first, outset of the disease will recover.

TREATMENT.—It is hardly worth while at the present time to speak upon the subject, and still, perhaps, one word will not be out of place. Quinine, Mercury and Iodine have all been tried as curative remedies. We have given Quinine but observe no very positive effects. Iodine in form of Iod. Potass, and Bromine in form of Bromide of Potassium, are favorite remedies with the profession and possesses at least one important feature—they can scarcely be supposed to do any harm. Opiates would be excluded upon theoretical grounds, but the pain, restlessness and general distress is greatly relieved by anodynes, and we cannot withhold the conviction that they are unproductive of harm, and if carefully selected and appropriately given, answer admirably the most important therapeutical indication. In many cases anodynes cannot be omitted in any consistency with good care—the pain *must* be relieved. The value of medication is yet to be determined by careful and continous observation.

These remarks are based upon early and not very extensive observation, and no very positive opinions are formed. We are ready to change in our views as facts may indicate, but most sincerely trust that our opportunities in Buffalo for studying this disease may be very limited.

We have not spoken a word as to the nature of the disease, as we have had no opportunities to observe the pathological changes which take place in fatal cases.

P. S. Since writing the above, we have seen one *post mortem* examination of a private patient of ours in the General Hospital, who died with well marked symptoms of the disease. The sections were made with great care and skill by the House Physicians and Surgeons, Drs. Fuller and Harrington, and showed the membranes of the brain and spinal cord very beautifully, but we are sorry to add the appearances were normal, or so nearly so, as to throw no light at all upon the causes of death. Examination was also made of all the organs of the Chest and Abdomen and no pathological changes were anywhere observed.

